# Curcumin Exerts its Anti-hypertensive Effect by Down-regulating the AT_1_ Receptor in Vascular Smooth Muscle Cells

**DOI:** 10.1038/srep25579

**Published:** 2016-05-05

**Authors:** Yonggang Yao, Wei Wang, Meixiang Li, Hongmei Ren, Caiyu Chen, Jialiang Wang, Wei Eric Wang, Jian Yang, Chunyu Zeng

**Affiliations:** 1 Department of Cardiology, Daping Hospital, The Third Military Medical University, Chongqing, P. R. China; 2 Chongqing Institute of Cardiology, Chongqing, P. R. China; 3 Department of Nutrition, Daping Hospital, The Third Military Medical University, Chongqing, P. R. China

## Abstract

Curcumin exerts beneficial effects on cardiovascular diseases, including hypertension. However, its mechanisms are unknown. We propose that curcumin prevents the development of hypertension by regulating AT_1_ receptor (AT_1_R) expression in arteries. The present study examined how curcumin regulates AT_1_R expression in vascular smooth muscle cells and investigated the physiological significance of this regulation in angiotensin (Ang) II-induced hypertension. The results showed that curcumin decreased AT_1_R expression in a concentration- and time-dependent manner in vascular smooth muscle cells. Using luciferase reporters with an entire AT_1_ or a mutant AT_1_R in A10 cells, the AT_1_R promoter activity was inhibited by 10^−6 ^M curcumin, and the proximal element (from −61 to +25 bp) of the AT_1_R promoter was crucial for curcumin-induced AT_1_R down-regulation. An electrophoretic mobility shift assay showed that curcumin decreased specificity protein 1 (SP1) binding with the AT_1_R promoter in A10 cells. Curcumin treatment reduced Ang II-induced hypertension in C57Bl/6J mice, which was accompanied by lower AT_1_R expression in the arteries and decreased Ang II-mediated vasoconstriction in the mesenteric artery. These findings indicate that curcumin down-regulates AT_1_R expression in A10 cells by affecting SP1/AT_1_R DNA binding, thus reducing AT_1_R-mediated vasoconstriction and subsequently prevents the development of hypertension in an Ang II-induced hypertensive model.

The renin-angiotensin system (RAS) is one of the most important regulators of arterial blood pressure, and RAS activation is related to the pathogenesis of hypertension^1^. Angiotensin II (Ang II), the principal RAS effector peptide, binds to two distinct receptors: the Ang II type-1 receptor (AT_1_R) and the Ang II type-2 (AT_2_R) receptor. Most Ang II actions are transmitted via AT_1_R, including vasoconstriction, reduction of vascular compliance, cardiac contractility, cellular dedifferentiation and proliferation. Therefore, AT_1_R antagonists decrease blood pressure in patients with hypertension^2^,^3^.

Turmeric (prepared from the root of *Curcuma longa*) belongs to the ginger family. Curcumin, which is the main active constituent of turmeric, exerts anti-oxidant, anti-bacterial, anti-fungal, anti-viral, anti-inflammatory, anti-proliferative, pro-apoptotic and anti-atherosclerotic effects. Curcumin has been used in traditional Indian medicine for the treatment of many diseases, such as wounds, arthritis, trauma, ulcers, fever and skin diseases, over the past 60 years^4^,^5^. Furthermore, curcumin exerts beneficial effects on cardiovascular disease, although the molecular targets are multiple. Curcumin might regulate DNA polymerase, protein kinase C, lipoxygenase, growth factors, transcription factors, inflammatory cytokines, adhesion molecules and apoptosis-related proteins^6^. Recently, curcumin was reported to prevent the development of hypertension and vascular remodeling and to protect against cadmium-induced vascular dysfunction and hypertension^7^,^8^,^9^,^10^. We propose that curcumin might prevent the development of hypertension by regulating AT_1_R in arteries. The present study examined how curcumin regulates AT_1_R expression in A10 cells, a rat thoracic aorta-derived smooth muscle cell line, and investigated the physiological significance of this regulation in Ang II-induced hypertension. The results shows that, via affecting specificity protein 1 (SP1)/AT_1_R DNA binding, curcumin down-regulated AT_1_R expression in A10 cells, reduced AT_1_R-mediated vasoconstriction, and subsequently prevented the development of hypertension in an Ang II-induced hypertensive model.

## Results

### The inhibitory effect of curcumin on AT_1_R expression at the transcriptional level in A10 cells

**Figure 1:**
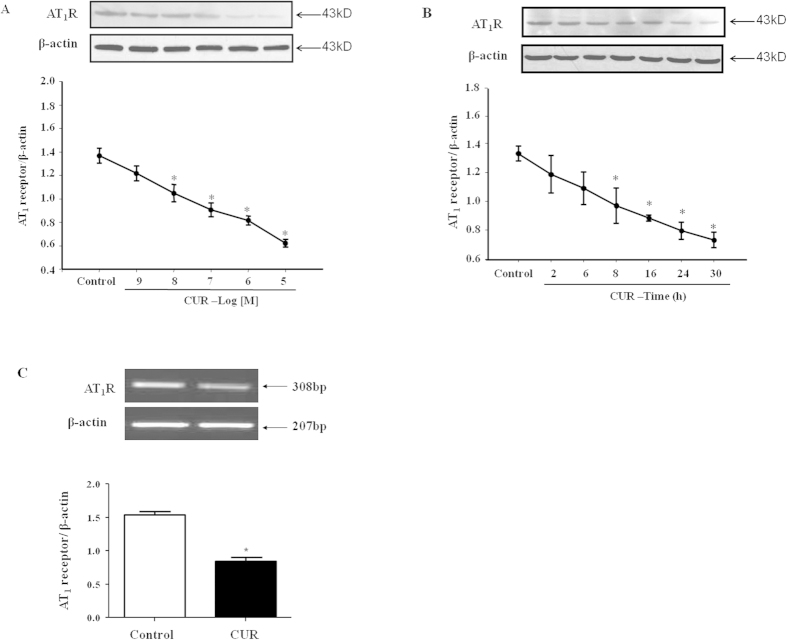
The inhibitory effect of curcumin on AT_1_R protein and mRNA expression in A10 cells. Curcumin decreased the AT_1_R protein expression in a concentration- (for 24 hrs) (**A**) and time- (at 10^−6 ^M) (**B**) dependent manner. (**C**) AT_1_R mRNA expression was also inhibited by curcumin (CUR, 10^−6 ^M/24 hrs) compared with the control (**C**). Cropped gels/blots were used in the figure; the AT_1_R expression was normalized against β-actin. The values are expressed as the mean ± SEM (*P < 0.05 vs. control, n = 6).

A10 cells were incubated with curcumin at different concentrations and for different time periods. Curcumin decreased the AT_1_R protein expression in a concentration (10^−5^–10^−9 ^M, 24 h)- and time (10^−6 ^M, 2–30 h)-dependent manner. Western blot analysis revealed that curcumin decreased AT_1_R protein levels significantly at 10^−8 ^mol/L compared with the control level, and the reduction reached a maximum at 10^−5 ^mol/L (Fig. 1A). Curcumin also down-regulated AT_1_R protein levels in a time-dependent manner; the reduction reached a maximum at 30 h (Fig. 1B). The expression of AT_1_R mRNA, as determined by RT-PCR, was significantly reduced by curcumin (10^−6 ^M) at 24 h compared with the control (Fig. 1C). Therefore, the regulation of curcumin on AT_1_R expression may occur at the transcriptional or post-transcriptional level.

**Figure 2:**
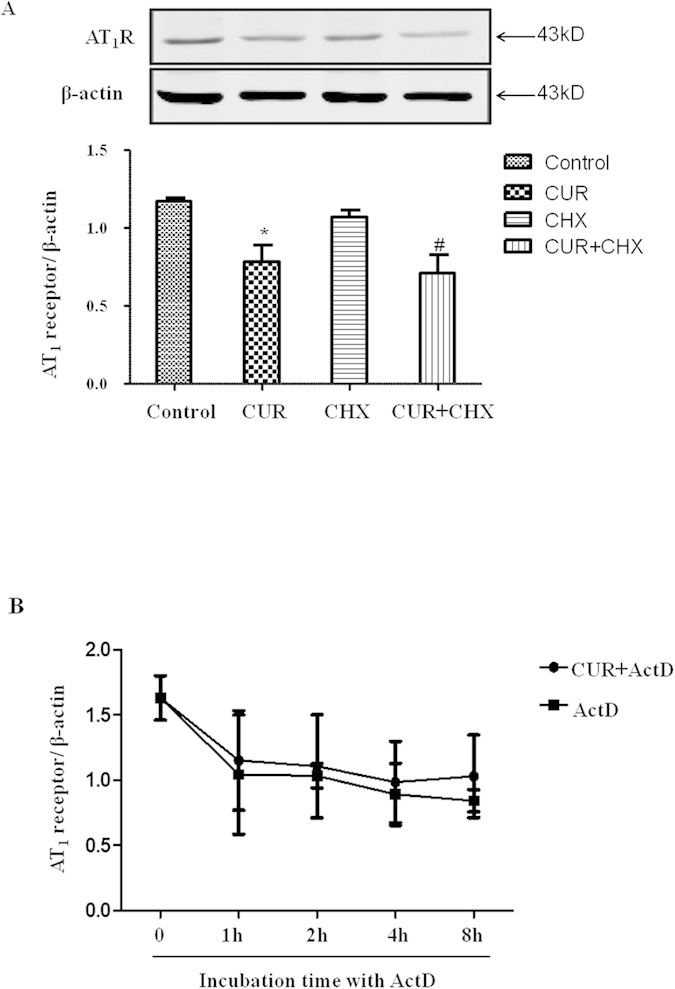
Effect of cycloheximide and actinomycin D on curcumin-induced AT_1_R downregulation. (**A**) The effect of cycloheximide (CHX) on curcumin (CUR)-induced AT_1_R protein down-regulation was examined by Western blot analysis; cropped blots are shown. The AT_1_R expression level was normalized against β-actin (*P < 0.05 vs. control, ^#^P < 0.05 vs. CHX, n = 7). (**B**) The effect of actinomycin D (ActD) on CUR-induced AT_1_R mRNA down-regulation was examined by RT-PCR analysis. The AT_1_R expression was normalized against β-actin. The values are expressed as the mean ± SEM (*P < 0.05 vs. control, n = 7).

To determine whether the regulation of curcumin on AT_1_R occurs at the protein degradation level, A10 cells were treated with cycloheximide (10^−6 ^M) to block de novo protein synthesis. Cycloheximide had no effect on the down-regulation of curcumin on AT_1_R protein expression (Fig. 2A), which demonstrates that AT_1_R degradation is not involved in the mechanism. We then examined whether curcumin affects the stability of AT_1_R mRNA. Actinomycin D (ActD, 5 μg/ml), an inhibitor of de novo mRNA synthesis, was used in this study. Actinomycin D had no effect on the down-regulation of curcumin on AT_1_R expression (Fig. 2B). These results indicate that curcumin does not regulate AT_1_R protein expression at the protein or mRNA degradation levels, suggesting that curcumin regulates AT_1_R expression at the transcriptional level.

### Role of the transcription factor SP1 in curcumin-induced down-regulation of AT_1_R expression in A10 cells

**Figure 3:**
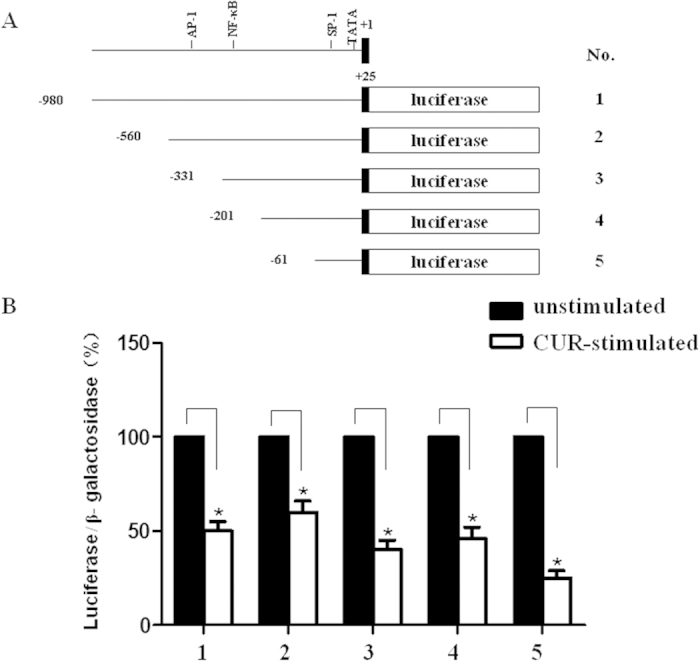
Effect of curcumin on AT_1_R mRNA transcription. (**A**) Schematic of deletion mutants of the AT_1_R promoter/luciferase fusion DNA construct. (**B**) The bar graphs indicate luciferase activity normalized against β-galactosidase activity derived from the corresponding deletion. The data indicate the luciferase activity of curcumin-stimulated A10 cells (white bars) relative to unstimulated (black bars) in each group. The values (mean ± SEM) are expressed as a percent (*P < 0.05 vs. unstimulated, n = 6).

To elucidate the molecular mechanism underlying the curcumin-induced down-regulation of AT_1_R gene expression, five AT_1_R promoter-luciferase DNA constructs were transfected into A10 cells (Fig. 3A). The deletion mutants of the AT_1_R promoter resulted in decreased promoter activity. AT_1_R promoter activity was inhibited by 10^−6 ^M curcumin in all constructs (Fig. 3B), suggesting that curcumin inhibited AT_1_R expression at the transcriptional level and that the proximal element (from −61 to +25 bp) of the AT_1_R promoter was crucial for curcumin-induced AT_1_R down-regulation.

**Figure 4:**
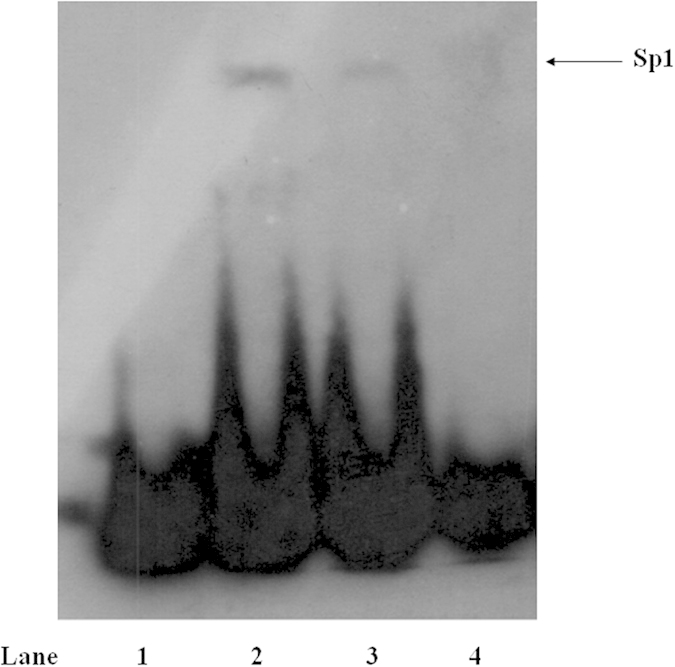
Effect of curcumin on the binding ability of SP1 at the AT_1_R gene promoter in A10 cells. The binding activity of the AT_1_R gene promoter (−40 bp to −6 bp), which contains an Sp1 site, was examined in the nuclear protein from unstimulated (lane 2) or curcumin (10^−6 ^M)-stimulated (lane 3) A10 cells by electrophoretic mobility shift assay; DNA-binding protein (arrow) was decreased compared with unstimulated A10 cells. No nuclear extracts or 50 times of unlabeled probe were added to the reaction mixture as controls (lanes 1 and 4, respectively).

The ubiquitous transcription factor SP1 is important for the regulation of AT_1_R expression^11^,^12^. We detected DNA-binding protein bound to the SP1 site using an electrophoretic mobility shift assay (Fig. 4). When nuclear extracts from curcumin-stimulated A10 cells (lanes 3) were used, DNA-binding protein (arrow) was decreased compared with unstimulated A10 cells (lanes 2). No nuclear extracts and 50 times of unlabeled probe were added to the reaction mixture as controls (lanes 1 and 4, respectively), and these controls did not exhibit this band, confirming the binding specificity.

### Inhibition of AT_1_R expression by Curcumin and its function in arteries from Ang II-induced hypertensive C57Bl/6J mice

**Figure 5:**
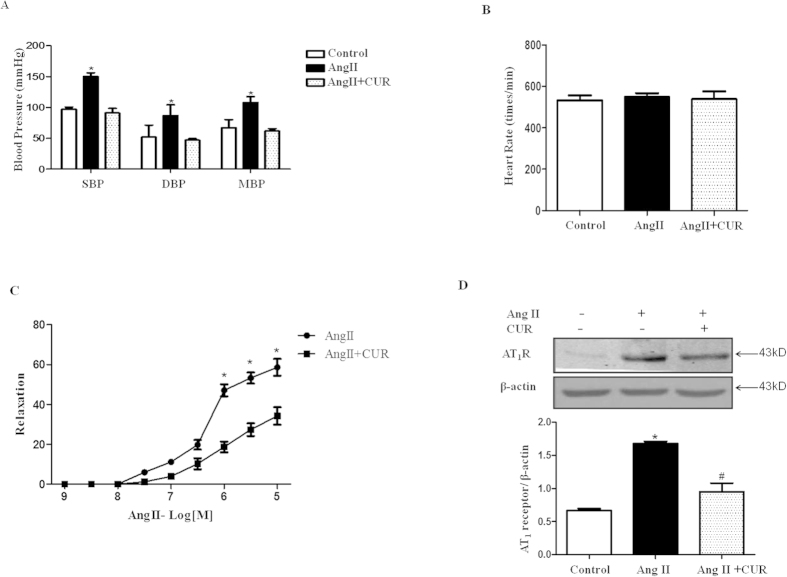
Effect of curcumin on AT_1_R expression and function in arteries from Ang II-induced hypertensive C57Bl/6J mice. The blood pressure (**A**) and heart rate (**B**) were examined in Ang II-induced hypertensive C57Bl/6J mice with or without curcumin treatment (*P < 0.05 vs. control, n = 5). The Ang II-mediated vasoconstriction in the third-order mesenteric arteries (**B**) and the AT_1_R expression in the aorta (**D**) were examined in these mice (*P < 0.05 vs. others, n = 7). Cropped blots were used in this figure; the AT_1_R expression was normalized against β-actin. The values are expressed as the mean ± SEM (*P < 0.05 vs. control group, #P < 0.05 vs. Ang II group, n = 7).

To investigate the physiological significance of the regulation of curcumin on AT_1_R expression, we established an Ang II-induced hypertension model in C57Bl/6J mice. Eight-week-old mice (weight 20 g ± 0.89) were divided into three groups: control, Ang II (490 ng/min/kg), and Ang II (490 ng/min/kg) + curcumin (300 mg/kg/d). Ang II was administered subcutaneously via an osmotic minipump (Model 1004, ALZET Osmotic Pumps, CA) for one week. Curcumin was suspended in normal saline, and the mice were given the suspension by oral gavage once per day; normal saline was used as a control. After one week, the blood pressure and heart rate were measured using the tail-cuff method. The blood pressures of the Ang II-treated mice (SBP: 150 ± 6.24 mmHg, DBP: 86 ± 17.61 mmHg) were significantly higher than the Ang II + curcumin group (SBP: 91 ± 7.55 mmHg, DBP: 47 ± 2.64 mmHg) (Fig. 5A). However, the heart rate was not significantly different between the individual groups (Fig. 5B).

To determine how curcumin exerts its function in Ang II-related hypertension, we studied AT_1_R function in third-order mesenteric arteries from the mice of the Ang II and Ang II + curcumin groups. Isolated mesenteric arteries from normal C57Bl/6J mice were used to measure the contractile responses to different concentrations of phenylephrine and KCl and the relaxant response to acetylcholine (Ach). The effect of curcumin (10^−6 ^M) alone on mesenteric arteries was compared. The mesenteric arteries were incubated with curcumin for 60 min before starting the experiment. The changes in contractile and relaxant responses to phenylephrine, KCl and Ach were also measured. Our results showed that curcumin slightly decreased the contractile response of aortic rings to phenylephrine and KCl (Supplemental Fig. 1A), whereas it did not affect the relaxant response to Ach (Supplemental Fig. 1B). We found that Ang II (10^−9^–10^−5 ^M) induced concentration-dependent vasoconstriction in the third-order mesenteric arteries from the mice; the vasoconstrictive effect of Ang II was lowered significantly by curcumin (Fig. 5C). Further experiments were performed to examine AT_1_R expression in the arteries, and curcumin reduced the increased AT_1_R protein levels in the aortas of Ang II-induced hypertensive C57Bl/6J mice (Fig. 5D).

## Discussion

Previous studies have demonstrated that curcumin possesses strong antioxidant and cardioprotective properties. Therefore, curcumin is considered to be a new therapeutic candidate for cardiovascular diseases, including idiopathic pulmonary arterial hypertension, glomerular hypertension and L-NAME-induced hypertension^7^,^8^,^9^,^10^,^13^,^14^. However, the role of curcumin in hypertension and its associated complications remains unknown. In this study, we determined that curcumin suppressed AT_1_R expression in *in vitro* and *in vivo* experiments. To our knowledge, this is the first report demonstrating the down-regulation of vascular AT_1_R by curcumin.

The beneficial effects of curcumin on L-NAME-induced vascular dysfunction are associated with increased eNOS protein expression, reduced oxidative stress, and replenished antioxidant glutathione with partial restoration of normal redox status^7^. As one of the major regulatory factors of blood pressure, we were interested in whether curcumin regulated AT_1_R expression. Our present study showed that curcumin lowers the blood pressure in Ang II-induced hypertensive C57Bl/6J mice, and this anti-hypertensive effect is partially attributed to the inhibition of AT_1_R expression in arteries. Moreover, curcumin-regulated AT_1_R expression in the arteries is of physiological significance because the Ang II-mediated vasoconstrictive effect is lower in Ang II-induced hypertensive C57Bl/6J mice after treatment with curcumin. The anti-oxidative effect of curcumin has been shown in hypertensive rats^7^,^10^,^15^,^16^; increased ROS increases AT_1_R expression in the kidney^17^,^18^,^19^. However, whether ROS is involved in the regulation of curcumin on arterial AT_1_R expression remains unknown and needs to be determined in the future.

The underlying mechanisms regulating AT_1_R expression are complicated and might involve several levels, including transcriptional and post-transcriptional levels^13^,^17^,^20^,^21^. To determine whether the regulation occurs at the post-transcriptional or post-translational levels, we treated cells with cycloheximide to block de novo protein synthesis or actinomycin D to block de novo mRNA synthesis; cycloheximide and actinomycin D had no effect on the regulation of curcumin on AT_1_R expression in A10 cells, which demonstrated that the regulation of curcumin on AT_1_R protein expression did not occur at the protein or mRNA degradation levels and that the regulation of curcumin on AT_1_R expression occurs at the transcriptional level. The deletion mutant analysis of the AT_1_R gene promoter (from −980 bp to +25 bp) revealed that the inhibition of AT_1_R expression by curcumin depends on the most proximal promoter region (from −61 bp to +25 bp), which contains an SP1 binding site (GC box). A gel shift assay showed that DNA-binding protein bound to the SP1 site was decreased in curcumin-stimulated A10 cells, indicating a crucial role of the SP1 site in curcumin-induced AT_1_R down-regulation.

This study demonstrated that curcumin suppressed AT_1_R protein expression by inhibiting SP1 activity. The ability of curcumin to inhibit AT_1_R expression might be of clinical significance. Whether or not there is a synergistic or additional effect between curcumin and other anti-hypertensive medicines, including ARBs and ACE inhibitors, needs to be identified in the future.

## Methods

### Materials

The rabbit polyclonal AT_1_R antibody was purchased from Santa Cruz Biotechnology (sc-1173, Dallas, TX). Curcumin was obtained from Sigma (c7727, St. Louis, MO); Ang II was purchased from Calbiochem (4474-91-3, San Diego, CA); Dulbecco’s modified eagle medium (DMEM, Ca# 11995065,) and fetal bovine serum (FBS, Ca# 16000044) were obtained from Life Technologies (Carlsbad, CA). All other chemicals for various buffers were of the highest purity available and were purchased from Sigma or Gibco (Grand Island, NY).

### Cell Culture

Embryonic thoracic aortic smooth muscle cells (passage 10–20) from normotensive Berlin-Druckrey IX^22^ (A10; American Type Culture Collection, Manassas, VA) were cultured in DMEM supplemented with 10% FBS at 37 °C in a 95% air-5% CO_2_ atmosphere and were used for the experiments.

### Western Blot Analysis

The expression of AT_1_R was detected by Western blot analysis, as described previously^23^. The specific band was scanned with an imaging analyzer, and β-actin was used as a loading control. The expression level of AT_1_R was indicated as a ratio of AT_1_R to β-actin.

### RT-PCR for AT_1_R

A total of 2 μg of total RNA extracted from A10 cells was used to synthesize cDNA and served as a template for the amplification of AT_1_R and β-actin, which served as the house-keeping gene control. The AT_1_R mRNA expression levels were normalized to β-actin mRNA. The primers for β-actin included forward primer 5′-CCACTGCCGCATCCTCTT-3′ and reverse primer 5′-GTCAGCAATGCCTGGGTA-3′ (GenBank accession No. NM_031144, 251 bp). The primers for AT_1_R included forward primer 5′-AAATTGAGTGGCTGTATG-3′ and reverse primer 5′-CTTGACCTCCCATCTCCT-3′ (GenBank accession No. NM_031009, 160 bp).

### Transfection of AT_1_R Promoter-Luciferase Fusion DNA Construct into VSMCs

Five deletion mutants of the rat AT_1_R gene promoter region were ligated to the luciferase gene, as described previously^24^. A10 cells were prepared in a 6-cm tissue culture dish. After 48 hours, 5 μg of the AT_1_R promoter luciferase fusion DNA construct and 2 μg of the LacZ gene driven by the SV40 promoter-enhancer sequence were transfected into the A10 cells. In addition to the AT_1_R promoter luciferase fusion DNA construct (980 bp, 2 μg) and the LacZ gene (2 μg), vector plasmid (3 μg) was introduced concomitantly into the A10 cells. These cells were stimulated with 10^−6 ^M^25^,^26^,^27^ curcumin and cultured in DMEM with 10% FBS for 48 hours. The luciferase activity was then measured and normalized against the β-galactosidase activity.

### Preparation of Nuclear Extracts and Electrophoretic Mobility Shift Assay

An EMSA was performed with a Light-shift Chemiluminescent EMSA Kit (Ca# 20158, Thermo Fisher Scientific, Waltham, MA) according to the manufacturer’s recommendations^28^. Using a non-isotopic method to detect DNA–protein interactions, this system includes an enhanced luminol substrate for horseradish peroxidase with optimized blocking and washing buffers that produce sensitivity equivalent to radioactive (^32^P) systems. A synthetic DNA double-stranded oligonucleotide probe containing the sequence of the rat AT_1_R gene promoter between nucleotides −40 and −6 bp (5′-GGAACCTGCAGAGCAGCGA CGCCCCCTAGGCT ATA-3′, 3′-CCTTGGACGTCTCGTCGCTGCGGGGGATCCGATAT-5′, both of which contain an Sp1 site) was labeled with biotin and incubated with the nuclear extracts. After the reaction, the DNA–protein complexes were subjected to 6% native polyacrylamide gel electrophoresis and transferred onto a nylon membrane (Millipore Corp. Lincoln Park, NJ). After transfer, the membrane was immediately cross-linked for 15 min on a UV transilluminator. A chemiluminescent detection method using a luminal/enhancer solution and stable peroxide solution (Thermo Fisher Scientific, Waltham, MA) was used, as described by the manufacturer, and the membranes were exposed to X-ray films for 30 s to 5 min before development.

### Animal Experiment

Eight-week-old C57Bl/6J mice, weighing approximately 20 g, were purchased from the Laboratory Animal Center of Daping Hospital of the Third Military Medical University. The mice were divided into the following experimental groups: control, Ang II (490 ng/min/kg), and Ang II (490 ng/min/kg) + curcumin (300 mg/kg/d). Curcumin was suspended in normal saline, and the mice were administered the suspension by oral gavage once per day for one week. The estimated dose of curcumin was 300 mg/kg/d^29^,^30^. In the Ang II group, 490 ng/min/kg of Ang II was administered subcutaneously via an osmotic minipump (Model 1004, ALZET Osmotic Pumps, CA). After one week, the blood pressure and heart rate were measured using the tail-cuff method (UR-5000, UEDA, Japan); the mice were then sacrificed under pentobarbital anesthesia (60 mg/kg). The thoracic aorta was removed quickly, minced into small pieces, lysed in a lysis buffer, sonicated, placed on ice for 1 h and centrifuged at 15000 rpm for 30 min at 4 °C. The supernatants were stored at −70 °C until being subjected to Western blot analysis for AT_1_R and β-actin.

All study procedures were approved by the Third Military Medical University Animal Use and Care Committee. All experiments conformed to the guidelines of the ethical use of animals, and all efforts were made to minimize animal suffering and to reduce the number of animals used.

### Mesenteric Artery Study

Each mouse was anesthetized with pentobarbital. The total mesenteric bed was removed carefully and placed in an ice-cold physiological salt solution (PSS, containing NaCl 119 mM, KCl 4.7 mM, CaCl_2_·H_2_O 2.5 mM, MgSO_4_·H_2_O 1.17 mM, NaH_2_CO_3_ 25 mM, KH_2_PO_4_ 1.18 mM, EDTA 0.027 mM, and glucose 5.5 mM, adjusted to pH 7.4). The surrounding fat and connective tissues of the mesenteric artery were carefully and quickly dissected. Third-order branches of the superior mesenteric artery were cut into rings approximately 2 mm in length and mounted on 25 μm tungsten wires in an isometric Mulvany-Halpern small-vessel myograph (model 91 M610, J.P. Trading, Science Park, Aarhus, Denmark). The rings were maintained in ice-cold PSS at 37 °C and continuously bubbled with oxygen (95%) and carbon dioxide (5%) (Carbogen). All procedures were performed carefully to protect the endothelium from any damage. In the first set of experiments, the rings were contracted with high-potassium PSS (KPSS, 70 mM) to obtain the maximal response^31^,^32^. After obtaining the maximal response to KPSS, the response curves to Ang II were measured by a cumulative concentration-dependent protocol (10^−9^ to 10^−5 ^M). To test the curcumin-induced inhibition of vasoconstriction, the mice were administered curcumin by oral gavage once per day for 1 week before the Ang II treatment.

### Statistical Analysis

Statistical analysis was performed with performed 1- or 2-way ANOVA and Fisher’s test, if appropriate. The data are shown as the mean ± SEM. A value of P < 0.05 was considered significant.

## Additional Information

**How to cite this article**: Yao, Y. *et al.* Curcumin Exerts its Anti-hypertensive Effect by Down-regulating the AT_1_ Receptor in Vascular Smooth Muscle Cells. *Sci. Rep.* **6**, 25579; doi: 10.1038/srep25579 (2016).

## Supplementary Material

Supplementary Information
