# Supplementary material for: Curcumin Exerts its Anti-hypertensive Effect by Down-regulating the AT1 Receptor in Vascular Smooth Muscle Cells
Source: Sci Rep. 2016 May 5;6:25579. doi: 10.1038/srep25579 (PMC4857140; doi:10.1038/srep25579)

**Supplementary Information**

**Curcumin Exerts Anti-hypertensive Effect by Downregulation of AT_1_ Receptor in Vascular Smooth Muscle Cells**

Yonggang Yao^1,2*^, Wei Wang^1,2*^, Meixiang Li^1,2^, Hongmei Ren^1,2^, Caiyu Chen^1,2^, Jialiang Wang^1,2^, Wei Eric Wang^1,2^, Jian Yang^1,2,3^, Chunyu Zeng^1,2^

**Supplementary** **Figure 1: Effect of curcumin in mesenteric arteries from C57Bl/6J mice.**

The mesenteric arteries from C57Bl/6J mice were incubated with curcumin before treatment with PHE or Ach (10-6 M). The contractile responses with curcumin are shown in **Supplementary Figure 1A** (*p<0.05 vs. control, n=6), and the relaxant responses are shown in **Supplementary Figure 1B** (p>0.05 vs. control, n=6).


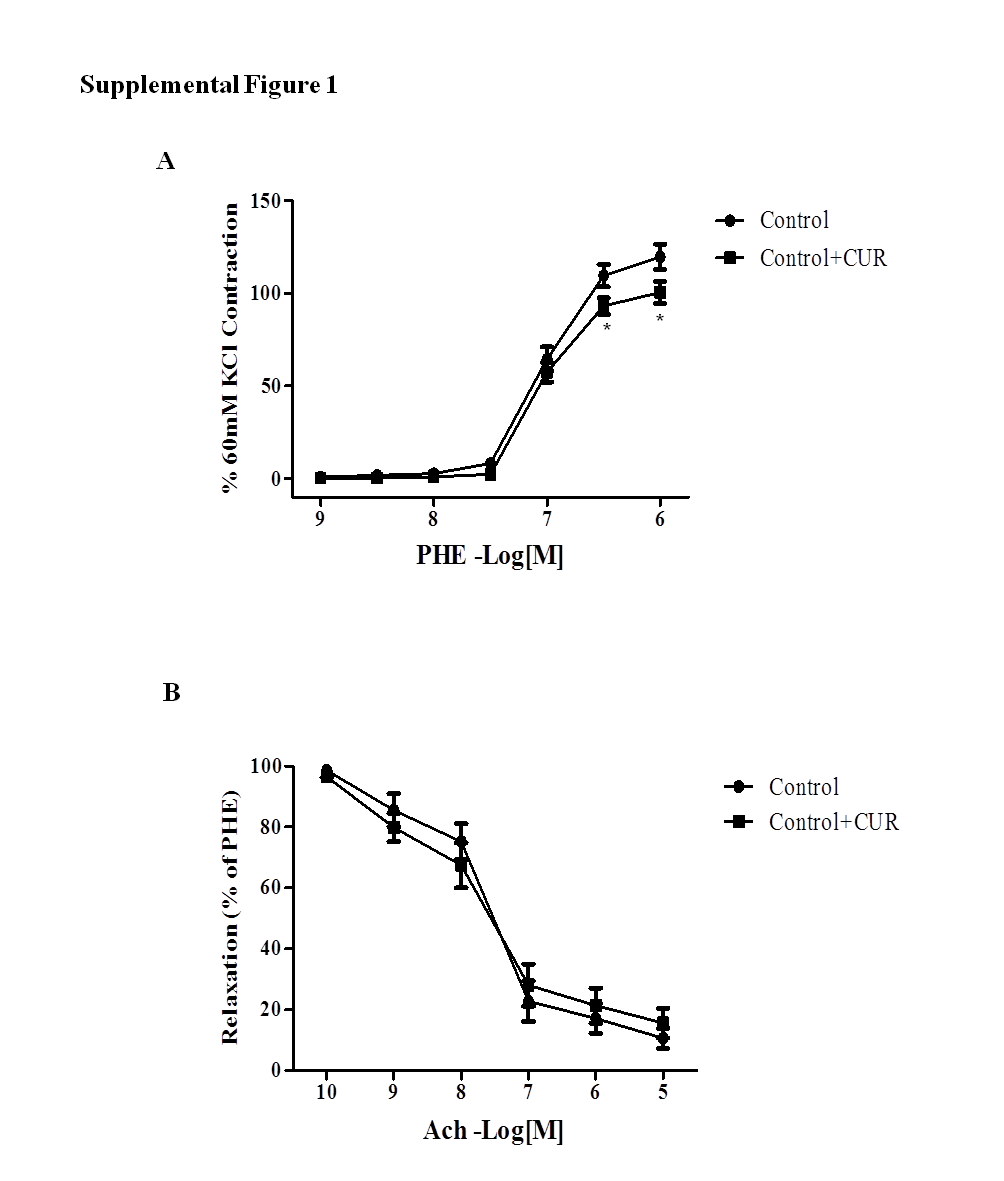


**Supplementary Figure 2. Full-length images of the immunoblots presented in the main figures.**


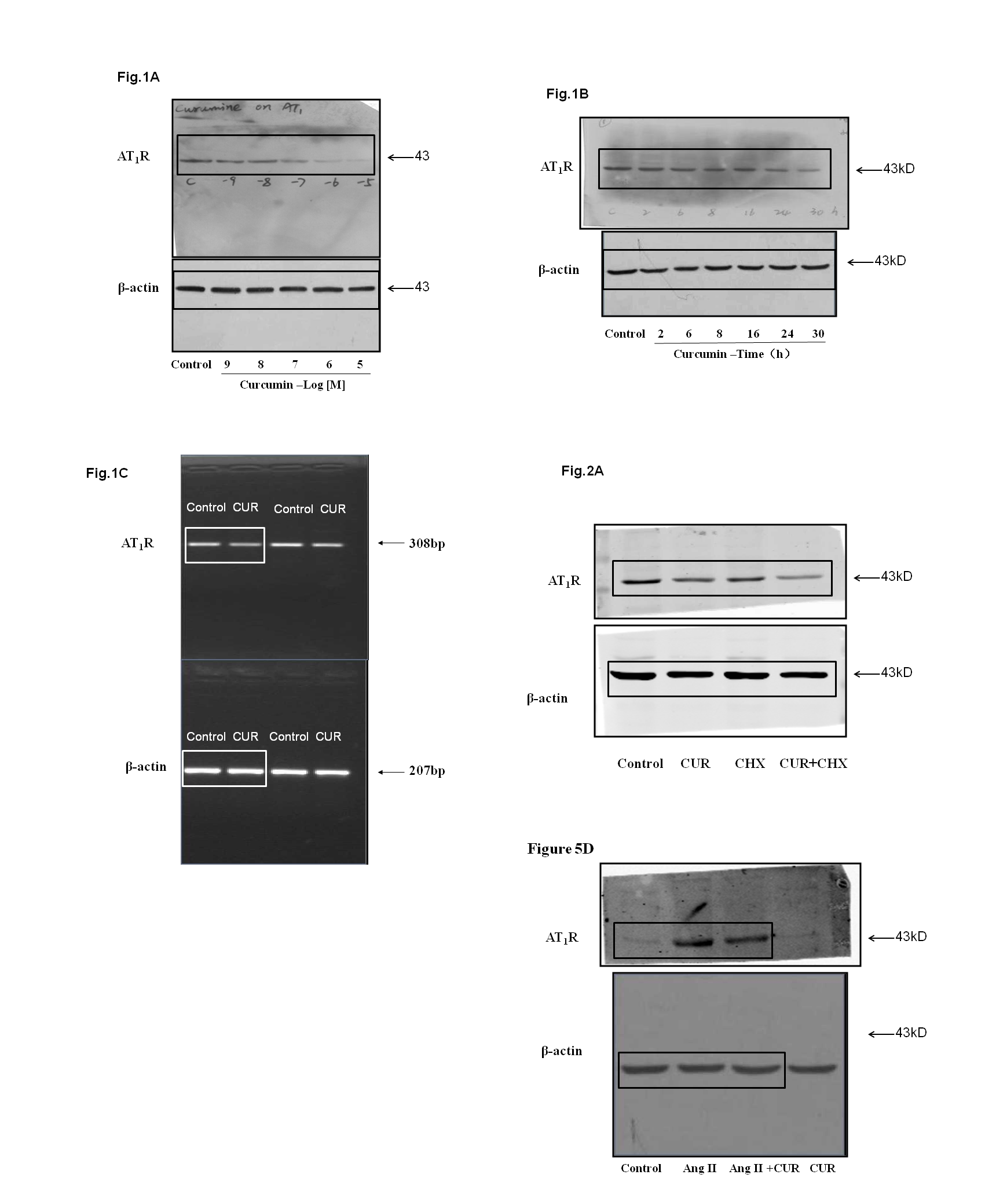

Supplement: Supplementary Information [file srep25579-s1.doc]
